# Genetic Structural Differentiation Analyses of Intercontinental Populations and Ancestry Inference of the Chinese Hui Group Based on a Novel Developed Autosomal AIM-InDel Genotyping System

**DOI:** 10.1155/2020/2124370

**Published:** 2020-08-25

**Authors:** Tong Xie, Chunmei Shen, Xiaoye Jin, Qiong Lan, Yating Fang, Bofeng Zhu

**Affiliations:** ^1^Multi-Omics Innovative Research Center of Forensic Identication, Department of Forensic Genetics, School of Forensic Medicine, Southern Medical University, Guangzhou 510515, China; ^2^Institute of Brain and Behavioral Science, College of Life Sciences, Shaanxi Normal University, Xi'an, China; ^3^Key Laboratory of Shaanxi Province for Craniofacial Precision Medicine Research, College of Stomatology, Xi'an Jiaotong University, Xi'an, China; ^4^Clinical Research Center of Shaanxi Province for Dental and Maxillofacial Diseases, College of Stomatology, Xi'an Jiaotong University, Xi'an, China

## Abstract

In the present study, we investigated the genetic polymorphisms of 39 ancestry informative marker-insertion/deletion (AIM-InDel) loci in the Chinese Hui group using a previously self-developed panel, further clarified the genetic relationships between the Hui group and other reference populations, and assessed the ancestry inference efficiency of the AIM-InDel panel based on the worldwide population data from 1000 Genomes Phase 3. The results of the locus-specific informativeness (*I*_n_) and pairwise fixation index (*F*_st_) values, multidimensional scaling analysis, and success ratio of estimation with cross-validation showed that the novel panel could well reveal the genetic structural differentiations of the East Asian, European, African, and South Asian populations. Besides, the biogeographical ancestry origin inference both at the individual and population levels was conducted on the Chinese Hui group by principal component analysis and STRUCTURE analysis, and the results revealed that the Hui group had the East Asian origin, and the East Asian component ratio of Hui group was approximately 88.87%. Furthermore, the population genetic analyses among the Hui group and reference populations were performed based on the insertion allele frequency heat map, population pairwise *F*_*st*_ values and phylogenetic tree, and the results indicated that the Hui group was genetically closer to East Asian populations, especially two Chinese Han populations (CHS and CHB populations).

## 1. Introduction

In recent years, ancestry informative inference has uncovered important information and provided a new perspective in biomedical fields such as anthropological research, forensic genetic application, and genetic epidemiology study [1–3]. In particular, ancestry inference based on ancestry informative genetic markers could also help to correct for population stratification [4–6]. Genetic variation describes the genotypic differentiations between different individuals or populations at the genomic DNA level, which was resulted from genetic mutation in connection with genetic drift, natural selection, and so on. The accumulation of genetic differences among populations, especially for intercontinental populations, is the basis of individual ancestor information inference. In the forensic genetic field, ancestry information inference could provide the valuable clue to the criminal case when the traditional genetic markers for individual identification failed to indicate the suspect. Currently, there were still some new challenges that need to be solved in ancestry inference research, such as elucidating the genetic variation estimations within or between populations and clearing the admixture proportions in an individual of mixed origin [7].

In the past decade, several panels on single nucleotide polymorphisms (SNPs) were developed for ancestry informative inference applications based on capillary electrophoresis (CE) platform, as well as massively parallel sequencing (MPS) technology [8, 9]. SNPs showed the advantages of favorable stability, widespread distributions, and relatively polymorphic allele frequency patterns in different populations [10], but several limitations (for example, SNP genotyping is a relatively complicated process and demands for a high-quality research platform) still existed in ancestry informative marker-single nucleotide polymorphism (AIM-SNP) analysis [11]. As for the mitochondrial DNA and Y chromosome genetic markers, although they separately possess highly ancestral information of the maternal and paternal inheritances, there are usually both no gene recombinations in these two genetic markers, and their variations show only the maternal or paternal genetic characteristics, respectively. Besides, the databases of these two kinds of genetic markers are limited; sometimes, it may lead to the deviations in genetic population analysis.

InDel is proposed as a new kind of genetic marker which combines the advantages of both short tandem repeats (STRs) and SNPs, i.e., extensive distribution, short amplicon size, and low mutation rate; besides, the length polymorphic characteristic makes it easy to be genotyped on the CE platform by fragment size differentiations [12, 13]. Another advantage of the InDel maker is the simple genotyping workflow which could reduce the risk of DNA contamination and save the genotyping time to a great extent. Compared with the AIM-SNP typing method based on SNaPshot technology, the technology of labeling the InDel primers by multicolor fluorescence materials, and combining with the CE platform, has the advantages of easy popularization and forensic application in the primary DNA laboratory [11]. Although MPS technology has provided a very effective genotyping method to simultaneously detect hundreds of genetic markers [14], it still required unified standards to make MPS technology as a routine method in forensic application. Hence, developing the small-scale ancestry information marker sets for a universally applicable CE analysis system is still needed. In the consideration of the superiorities of the InDel marker, a 39 autosomal AIM-InDel panel was developed in our previous study [15]. In the present study, the effectiveness evaluation of this panel was extended to further analyze the populations from the five intercontinental regions (Africa, Europe, South Asia, East Asia, and America).

China is a multiethnic country with 56 populations, and the Hui group is one of the largest ethnic minorities which lives in Chinese many regions such as the Ningxia, Gansu, Qinghai, Xinjiang, Henan, Anhui, Liaoning, Heilongjiang, and Shaanxi provinces. There were few previously genetic polymorphic studies of different genetic markers on the Hui group, so the Hui group was chosen as the research object in this study. Genetic evidences of ancestry inference markers such as SNPs indicated that the Hui group had closer genetic relationships with East Asian populations [16]. But to this day, the ancestry informative component of the Hui group is still unclear. And the present study is aimed at exploring the Hui group's genetic background and revealing the ancestral components of the Hui group based on this self-developed 39 AIM-InDel panel.

## 2. Materials and Methods

### 2.1. Sample Collections and Population Data Filtration

In this study, the 509 adults of the Hui group who lived in the Xinjiang Uyghur Autonomous region were involved, and all the volunteers who had given their written informed consents were healthy, unrelated, and selected from the local Hui group randomly. The collection procedure of all the samples was conducted under the human and ethical research principles of Southern Medical University and Xi'an Jiaotong University Health Science Center.

Besides, the reference population data were from the 1000 Genomes Phase 3 [17], and the detailed information of 26 reference populations (a total of 2504 individuals) in the five intercontinental regions (Africa, Europe, East Asia, South Asia, and America) was shown in Supplementary Table 1.

### 2.2. Sample Genotyping Using the 39 AIM-InDel Panel

In the present study, 509 DNA samples were prepared and amplified using the novel 39 AIM-InDel directed amplification kit without the DNA extraction step, and the PCR amplifications were conducted using the GeneAmp PCR System 9700 (Applied Biosystems, Foster City, USA) with the total 25 *μ*l volume of the reaction system, and all the reagent dosages as well as PCR reaction condition were performed according to the previous study [15]. The AIM-InDel PCR products were separated and detected by the CE platform using the ABI 3500 xL Genetic Analyzer (Applied Biosystems, Foster City, USA). The 39 AIM-InDel genotyping was performed by GeneMapper ID-X software version 1.5 (Applied Biosystems, Foster City, USA). In order to ensure the accuracy of AIM-InDel genotyping results, a negative control and positive control (9947A) and allelic ladder were involved in the experimentation.

### 2.3. Multiple Statistical Analyses

The allele frequencies, forensic parameters, and *P* values for Hardy–Weinberg equilibrium (HWE) tests of 39 AIM-InDel loci in the Hui group were calculated by the STRAF online program (version 1.0.5) [18]. Since the rs3034941 locus was excluded due to the lack of population genotype data in 1000 Genomes Phase 3, the raw genotype data of the same 38 AIM-InDel loci of the 2504 individuals from 26 worldwide populations were obtained. The pairwise *F*_st_ values of five intercontinental populations in pairs, herein, the same intercontinental populations as a whole, were assessed using by Arlequin software (version 3.5) on the basis of 38 InDel loci, respectively. The success ratio of population origin with cross-validation estimation, the population-specific divergence (PSD) values, and the principal component analysis (PCA) of the same 38 AIM-InDel loci among the different populations were performed in the online Snipper software (version 2.5) (http://mathgene.usc.es/snipper/analysispopfile2_new.html), and the informativeness (*I*_n_) values which also called Rosenberg's *I*_n_ values were calculated by the PSD values multiplied with 0.693, i.e., converting the natural log to log(2) [19, 20]. The multidimensional scaling (MDS) analysis [21] was conducted by SPSS software (version 20.0). Population genetic structure analysis among the Hui group and reference populations was calculated by STRUCTURE software (version 2.3.4) with the length of burn-in period 10,000 times followed by 10,000 MCMC repetitions [22]. Besides, the optimal *K* value was determined by the online software Harvester program (http://taylor0.biology.ucla.edu/structureHarvester/). The bar plots based on the results of STRUCTURE analysis were conducted by DISTRUCT software (version 1.1) [23]. And the analysis for pairwise *F*_st_ values based on the same 38 InDel loci among 22 worldwide populations (American populations excluded) and the Hui group were assessed using Genepop software (version 4.0). The pairwise *D*_A_ distances of the above populations were conducted by DISPAN software, and the phylogenetic tree was conducted using MEGA software version 7.0 on the basis of population pairwise *D*_A_ distances. The box plot conducted based on Rosenberg's *I*_n_ values, the heat maps (one insertion allele frequency heat map and two *F*_st_ heat maps), and the scatter diagram of MDS analysis were drawn by *R* software (version 3.4.4).

## 3. Results

### 3.1. Ancestral Information Inference Synthetic Evaluation of the Novel AIM-InDel Panel

In the present study, the ancestry inference synthetic efficiency and forensic practicability of this novel panel were conducted by assigning the population genotype data of the same 38 AIM-InDel loci in the 2504 worldwide individuals from the 1000 Genomes Phase 3, and the pairwise *F*_st_ and locus-specific Rosenberg's *I*_n_ values, the cross-validation estimation success ratios, and the MDS analysis were involved in these populations.

**Figure 1:**
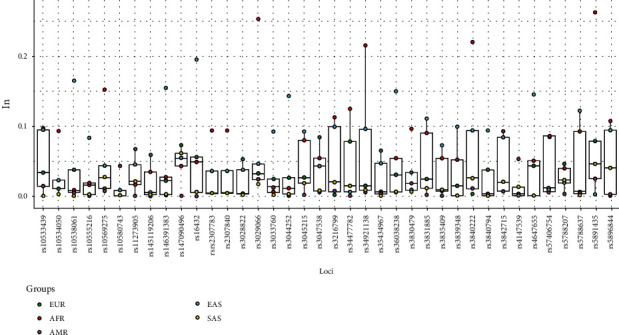
The box plot of locus-specific *I*_n_ values based on the same 38 AIM-InDel loci in five intercontinental populations from 1000 Genomes Phase 3.

The PSD values of all the AIM-InDel loci were calculated by the online software Snipper, and then, these values were converted to the more widely used Rosenberg's *I*_n_ values [19, 20]. As shown in Figure 1, the box plot of *I*_n_ values at the same 38 AIM-InDel loci showed distribution differences in five intercontinental populations from 1000 Genomes Phase 3, and the essential information of the total 39 AIM-InDel loci and Rosenberg's *I*_n_ values of the same 38 AIM-InDel loci in five intercontinental populations were shown in Supplementary Table 2. In the box plot, eight AIM-InDel loci (rs10538061, rs146391383, rs16432, rs3044252, rs36038238, rs3831885, rs4647655, and rs5788637) showed higher *I*_n_ values (>0.1) in East Asians; and eight AIM-InDel loci including the rs10569275, rs3029066, rs3216799, rs34477782, rs34921138, rs3840222, rs5891435, and rs5896844 could be regarded as African-informative markers with higher *I*_n_ values (>0.1) in Africans. As for Europeans, seven loci, i.e., rs11273905, rs147090496, rs3047538, rs34477782, rs35434967, rs57406754, and rs5891435 showed relatively higher *I*_n_ values (>0.06), which contributed greatly to differentiate the European populations and other intercontinental populations. In this panel, the locus-specific *I*_n_ values of South Asians and Americans were relatively lower than those of other three intercontinental populations mentioned above.

**Figure 2:**
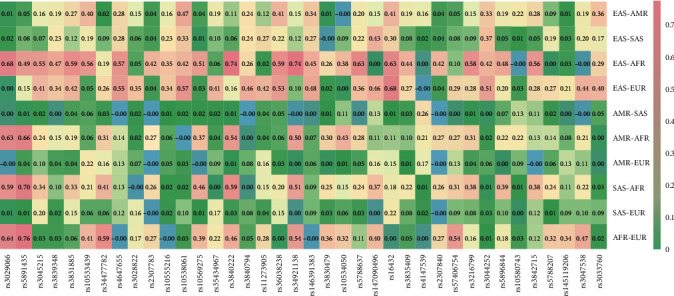
Pairwise *F*_st_ values at the same 38 AIM-InDel loci among pairwise intercontinental populations from 1000 Genomes Phase 3.

The pairwise *F*_st_ values were calculated among five intercontinental populations in pairs by the Arlequin software, and the pairwise *F*_st_ value results were reflected by a heat map conducted by *R* software. A heat map of pairwise *F*_st_ values in the five intercontinental populations was shown in Figure 2, and the pairwise *F*_st_ values were represented by different depths of various colors in these grids. When *F*_*st*_ values over 0.25, there were 10 loci in the EAS-AMR pair, 7 loci in the EAS-SAS pair, 27 loci in the EAS-AFR pair, 24 loci in the EAS-EUR pair, only one locus in the AMR-SAS pair, 13 loci in the AMR-AFR pair, 15 loci in the SAS-AFR pair, and 17 loci in the AFR-EUR pair, whereas there was no locus with the *F*_st_ value over 0.25 in the AMR-EUR pair and SAS-EUR pair.

The success ratios of population origin estimation with cross-validation of this panel were calculated using the Snipper software, and the results were shown in Table 1. In the AIM-InDel panel, three out of five intercontinental populations had the success ratios of ancestral information assignments over 90%, i.e., 98.49% (Africans), 91.25% (Europeans), and 99.80% (East Asians), while the South Asian and American populations represented relatively lower proportions for 84.67% and 61.96%, respectively.

**Figure 3:**
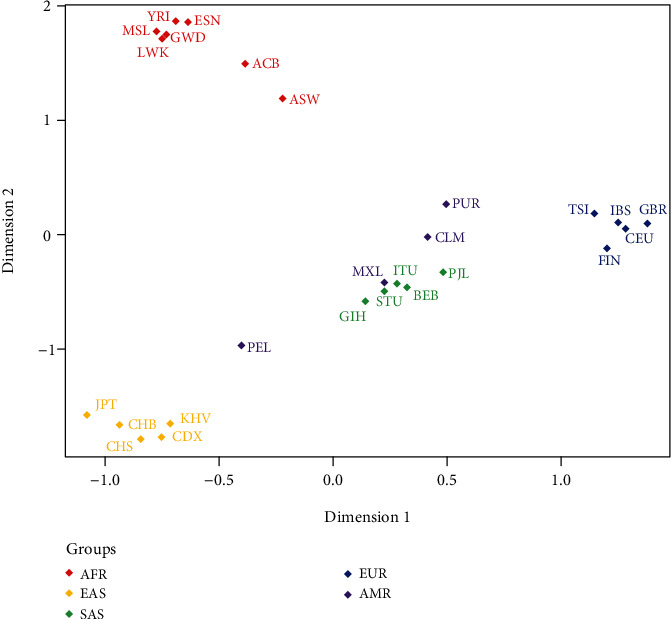
MDS analysis of the 26 populations from five continents based on allele frequencies of the same 38 AIM-InDel loci.

The MDS analysis of five different intercontinental populations was conducted on the population level via SPSS software, and the MDS result was shown in Figure 3. The multivariable relationships of 26 reference populations were represented in a two-dimensional scatter plot; each dot represented one population, and different colors were provided on behalf of different intercontinental populations. As for the discernibility effectiveness of this panel, the African, South Asian, East Asian, and European populations exhibited distinct clusters, respectively. And the populations from the same continent gathered together in the abovementioned four intercontinental populations, and separated from the other three intercontinental populations, whereas four American populations scattered around the South Asian clusters.

### 3.2. Ancestry Inference of the Hui Group Performed by a set of AIM-InDel Loci

The allelic frequencies and forensic parameters of the total 39 AIM-InDel loci in the Hui group were shown in Table 2. And the HWE tests for 39 loci were conducted as well; there were no significant deviations after the Bonferroni correction at all loci. The insertion allele frequencies were a range from 0.0285 (rs5896844) to 0.9293 (rs146391383) with the mean value of 0.5196. The matching probability, power of discrimination, polymorphic information content, power of exclusion, typical paternity index, observed heterozygosity, and expected heterozygosity of the 39 AIM-InDel loci ranged from 0.3539 (rs11273905) to 0.8992 (rs5896844), 0.1008 (rs5896844) to 0.6461 (rs11273905), 0.0538 (rs5896844) to 0.3748 (rs5788207), 0.0022 (rs5896844) to 0.2272 (rs5788207), 0.5258 (rs5896844) to 1.0923 (rs5788207), 0.0491 (rs5896844) to 0.5422 (rs5788207), and 0.0554 (rs5896844) to 0.5002 (rs5788207), with the mean values of 0.5017, 0.4983, 0.2850, 0.0967, 0.7864, 0.3405, and 0.3564, respectively.

**Figure 4:**
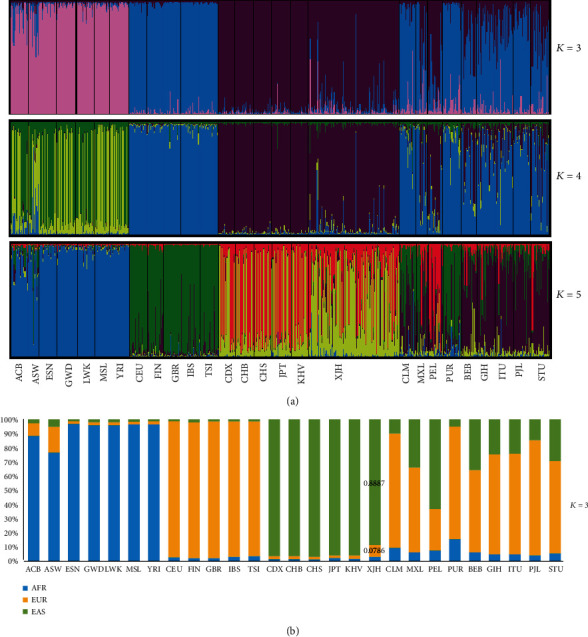
The population structure analyses and ancestry components of 26 populations at the individual (a) and population (b) levels, respectively.

In order to explore the ancestry components of the Hui group, population genetic structure analysis was conducted by STRUCTURE software based on the 26 reference populations. Firstly, the bar plots were conducted based on the raw genotype data of the total 3013 individual samples at *K* = 2-7, herein, only shown at *K* = 3-5. In Figure 4(a), when *K* = 3, the African populations were occupied mostly with color pink, European populations were almost blue, and East Asian populations were purple, but the American and South Asian populations showed mixed colors with blue and purple. The Hui group was accordant with East Asian populations which occupied mostly with color purple. When *K* = 4 and 5, the Hui group was still consistent with the ancestry information components with East Asian populations, while the American and South Asian populations could be distinguished with each other to a certain extent. The optimum *K* value was considered based on both the biogeographical factor and the result of delta *K* calculated by the online software Harvester program on the basis of the same 38 InDel loci in the total 27 populations from five different intercontinental populations, and the *K* value was finally determined at 3. As shown in Supplementary Table 3, when *K* = 3, the Hui group showed the ratios of ancestral informative components with the values of 0.8887 of cluster 1, 0.0786 and 0.0327 of cluster 2 and cluster 3, respectively, which were very similar to those of East Asian populations. The present study further assumed the Europeans, East Asians, and Africans as the three main ancestral origins to explore the ancestry proportions of unknown individuals and populations. As shown in Figure 4(b), the results were conducted on the population level, and the Hui group shared a relatively higher East Asian ancestry proportion (88.87%).

**Figure 5:**
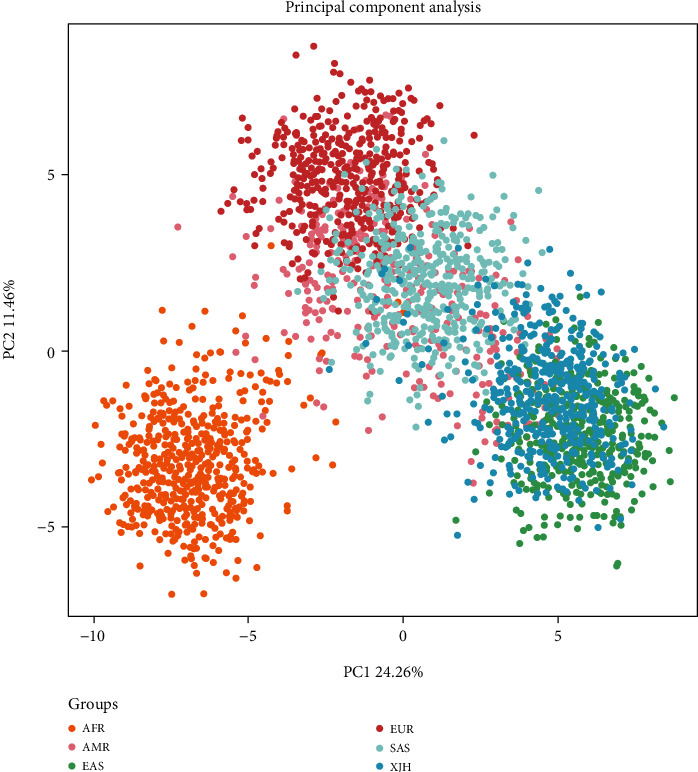
A scatter plot for PCA made by the online software Snipper based on raw genotype data of the total 3013 individuals from 27 populations.

A scatter PCA plot of the total 3013 individuals from 27 populations in five continents was conducted at the individual level by the online software Snipper based on raw genotype data of the same 38 AIM-InDel loci. As shown in Figure 5, only Hui individuals were labeled by the dark blue, but other individuals from five intercontinental populations were marked in five different colors according to their located continents. All the individuals except Americans were clustered into four respective main clusters, and almost all Americans were scattered between the European, East Asian and South Asian clusters. As for the studied Hui group, almost of the Hui individuals were scattered into the East Asian cluster, whereas few of which overlapped with the American and South Asian clusters.

### 3.3. Population Genetic Analyses of the Hui Group and Other Reference Populations via Multiple Methods

**Figure 6:**
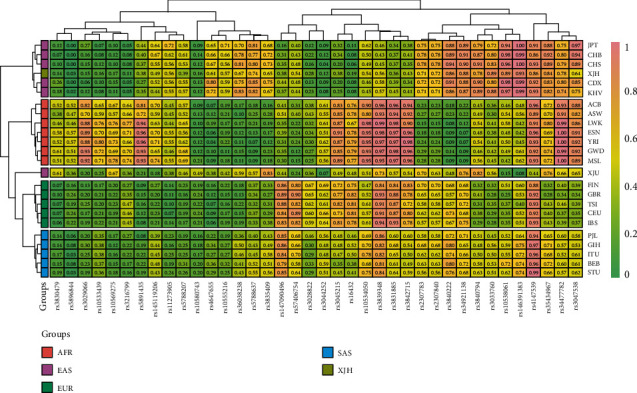
A heat map of insertion allele frequencies at the same 38 AIM-InDel loci among the Hui group and the reference populations drawn by R software.

The population genetic analyses were conducted among the Hui group and reference 22 populations from 1000 Genomes Phase 3 (American populations were excluded) and the reference Xinjiang Uyghur (XJU) group in our previous study [15]. The insertion allele frequencies of the same 38 AIM-InDel loci were compared among 22 different populations from four different intercontinental populations (African, European, East Asian, and South Asian), the XJU and Hui groups. As shown in Figure 6, the heat map intuitively displayed the insertion allele frequency distributions of 38 AIM-InDel loci by the different colors, which showed not only the genetic relationships of the total 24 different populations but also the clusters of 38 AIM-InDel loci. As shown in the heat map, the rs3028822, rs3044252, rs16432, and rs3045215 loci exhibited distinct lower insertion allele frequencies while the rs10538061, rs146391383, rs3840222, and rs34921138 loci showed relative higher insertion allele frequencies in East Asian populations. The rs5896844, rs3842715, rs3831885, rs10534050, rs3029066, and rs10569275 loci showed relatively higher insertion allele frequencies, whereas the rs2307783, rs2307840, rs3840222, and rs34921138 loci showed lower insertion allele frequencies in African populations. The insertion allele frequency distributions of the rs3044252, rs5788637, rs3835409, and rs36038238 loci ranged from 0.400 to 0.600 in South Asian populations. As for European populations, they showed relatively lower insertion allele frequencies in six loci rs35434967, rs34477782, rs3047538, rs3033760, rs10538061, and rs3840794 but higher insertion allele frequency values in three loci rs3028822, rs3044252, and rs57406754. On the whole, the 38 AIM-InDel loci in the heat map showed the frequency distribution differences in the four intercontinental populations. Meanwhile, the clustering relationships of four intercontinental populations were conducted as well, and the clustering results were consistent with the geographic distributions of their local continents; the Hui group was clustered with East Asian populations.

**Figure 7:**
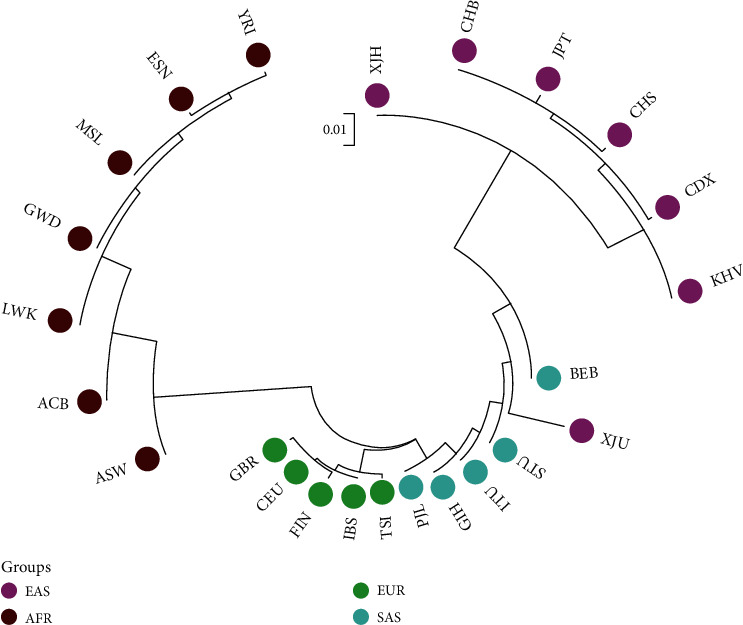
A phylogenetic tree constructed by the neighbor-joining method revealing the genetic relationships among the Hui group and the reference populations.

A phylogenetic tree was conducted based on pairwise *D*_A_ distances using the MEGA software, showing the genetic relationships among the Hui group and the other 23 reference populations. As shown in Figure 7, there were three main branches in the phylogenetic tree which included the African, European, and Asian branches, and herein, the populations in different continents were marked by different colors. As for Asian populations, the main branch could also be divided into two subbranches which included the East Asian and South Asian populations. The studied Hui group was located in the East Asian subbranch. The length of each branch represented the genetic distance between different populations. For further analyses, the Hui group had closer genetic relationships to the East Asian populations; oppositely, the largest genetic relationships were found among the Hui group and seven African populations.

**Figure 8:**
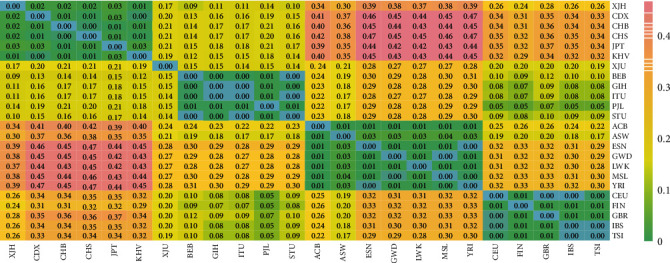
Interpopulation genetic analyses based on pairwise values among the Hui group and 23 reference population operated by Genepop software and R software.

In this study, the population pairwise *F*_st_ genetic distances were calculated among the 24 populations in pairs using the Genepop software, and a heat map intuitively represented the pairwise *F*_st_ value differences in Figure 8. As for the Hui group, the population pairwise *F*_st_ values ranged from 0.0116 (Hui-KHV) to 0.3885 (Hui-YRI). In the heat map, the pairwise *F*_st_ values were displayed by the different colors, and the blue color meant lower *F*_st_ values; however, the pink color represented higher *F*_st_ values. When considering the four main intercontinental populations, the East Asian populations showed the largest genetic differentiations with African populations, followed by the pairwise populations between the European and African populations. The studied Hui group had the smallest genetic differentiations with East Asian populations, especially with the KHV, CHB, and CHS populations, whereas it presented larger genetic differentiations with African populations such as the YRI, ACB, and LWK populations.

## 4. Discussion

This study chose the Xinjiang Hui group as the research object, and the Hui group is one of the largest ethnic minorities in China that spread across several provinces. The Xinjiang province was an important region along the historic Silk Road, and the Hui group was documented as being descended from Silk Road travelers according to the records [24, 25]. Exploring the genetic background and migration history of the Hui group is helpful to understand the complex population history of Xinjiang province. In recent years, ancestral informative inference can usually be used to correct the effect of population stratification in a genome-wide association study and also be applied to forensic anthropological research. Especially in the field of forensic genetic application, it is still necessary to investigate the population genetic diversity, further clarify population structure and background, and explore the biogeographic ancestor of the individual to which the biological materials from the crime scene belonged. The ancestral information inference research is helpful to narrow the criminal investigation scope and provide very valuable directional clues for the case investigation in forensic application. The most of the previously published panels for forensic ancestral inference have provided the important information for ancestral inference to some extent, but there were still some defects; for example, the genotyping for some AIM panels was a relatively complex process or required a specific or expensive detecting platform, which was difficult to be widely popularized and applied in the primary-level forensic DNA labs. Compared with the ancestor informative SNP, mitochondrial DNA and Y chromosome genetic markers, the novel AIM-InDel panel established previously by ourselves has the advantages of simple typing process, multiple amplification and capillary electrophoresis platform, and high efficiency of ancestor inference. The ancestry informative estimation of the Hui group was analyzed both at the individual and population levels. The ancestral origin components of the Hui group were inferred by STRUCTURE software based on an admixture ancestry model, and the results revealed that the Hui group shared the relatively higher East Asian ancestry proportion (88.87%). The PCA could be applied to describe some tangle genetic data with several principal components [26], and the PCA results also confirmed that the Hui group had East Asian ancestry origin. Usually, *F*_st_ values could be also regarded as a measure of population differentiation [27]. And the phylogenetic tree was a branching diagram showing the evolutionary relationships based on similarities and differences in genetic characteristics [28]. Furthermore, the results of the phylogenetic tree and population pairwise *F*_st_ values were conducted to further support the above results.

It has been pointed out that the small-scale panels with highly ancestral informative genetic markers could achieve the same effect on ancestral inference efficiency as the system with a great many of loci [29, 30]. Therefore, our group independently developed the 39 AIM-InDel system in the previous research and evaluated its ancestral information inference efficiency at three main intercontinental populations (East Asian, European, and African). And we extended the estimation of ancestral information inference efficiency to five intercontinental populations in this study. First of all, the pairwise *F*_st_ analyses among five intercontinental populations in pairs and Rosenberg's *I*_n_ values in five intercontinental populations were calculated on the same 38 AIM-InDel loci, and the obtained results showed that most of the 39 loci in the AIM-InDel system had high discrimination ability in four intercontinental populations except the American populations. In addition, the MDS analysis and success ratios of estimation with cross-validation verified that the novel panel could give satisfactory results in the population stratification of four intercontinental populations, i.e., the African, East Asian, European, and South Asian populations. Although the American populations showed relatively lower success ratios (61.96%) of estimation with cross-validation and pairwise *F*_st_ values, it might be due to the genetic background or structure of these reference American populations themselves, rather than the AIM-InDel loci we chose. The previously reported researches indicated that American populations have mixed and complex ancestral origins due to extensive gene exchange and population migration [31, 32]. Therefore, the lower *I*_n_ values of some loci and the ancestral inference efficiency of the AIM-InDel panel in the American populations were largely due to the mixed ancestral origins of the American populations. In general, the 39 AIM-InDel panel developed by ourselves was an effective, practical, and easy-operated tool, which could be successfully used to infer the ancestral informative inference of five intercontinental populations except the relatively lower efficiency in American populations. At the same time, it could be well applied in the current forensic DNA laboratory.

Besides, many genetic studies conducted by different genetic markers such as STR, Y chromosome haplogroup, and HLA-DRB1 also revealed that the Hui group had closer genetic relationships with East Asian populations [33–35]. As for ancestral inference, the present result was relatively similar to the finding of He et al. [16] which claimed 96.34% of East Asian ancestry component in the Hui group based on AIM-SNPs. Of course, the current research is not enough yet; in order to comprehensively and deeply reveal the population genetic relationships in Xinjiang province, more groups in this region and more molecular genetic markers should be studied in the future.

## 5. Conclusion

In this study, we assessed the ancestral inference efficiency of a self-developed 39 AIM-InDel panel and also explored the ancestral components of the Hui group. Multiple statistical analyses were conducted in order to assess the efficiency and to validate ancestry inference of this novel AIM-InDel panel. And this panel showed the satisfactory distinctions in four intercontinental populations and could be applied in forensic genetic analysis, anthropological research, and genetic epidemiology. The results of ancestral inference and population genetic analyses revealed that the Hui group shared relatively higher East Asian ancestry proportion (88.87%) and was genetically closer to East Asian populations (especially CHS and CHB populations). As for the Chinese Hui group in different regions, to further reveal its genetic background and migration history, more reference populations need to be involved in our future study.

## Figures and Tables

**Table 1:** The comparisons of success ratios of population origin estimation with cross-validation using the same 38 AIM-InDel loci among intercontinental populations (the populations from the same continent studied here were considered a whole) from five continents.

Population origins	American	African	European	East Asian	South Asian
Population of American origin	61.96%	1.44%	23.63%	6.92%	6.05%
Population of African origin	1.21%	98.49%	0.30%	0.00%	0.00%
Population of European origin	4.77%	0.00%	91.25%	0.00%	3.98%
Population of East Asian origin	0.20%	0.00%	0.00%	99.80%	0.00%
Population of South Asian origin	9.61%	0.00%	5.11%	0.61%	84.67%

**Table 2:** Allelic frequencies and forensic parameters of 39 AIM-InDel loci in the Chinese Hui group (*n* = 509).

Loci	MP	PD	PIC	PE	TPI	Ho	He	*P*	Insertion	Deletion
rs3029066	0.5912	0.4088	0.2197	0.0468	0.6715	0.2554	0.2515	0.8400	0.1473	0.8527
rs5891435	0.3913	0.6087	0.3612	0.1685	0.9568	0.4774	0.4736	0.8632	0.3841	0.6159
rs3045215	0.3828	0.6172	0.3590	0.1430	0.8993	0.4440	0.4693	0.2522	0.3752	0.6248
rs3839348	0.3900	0.6100	0.3708	0.1989	1.0262	0.5128	0.4921	0.3504	0.5648	0.4352
rs3831885	0.3974	0.6026	0.3554	0.1591	0.9357	0.4656	0.4626	0.8924	0.3625	0.6375
rs10533439	0.5706	0.4294	0.2367	0.0435	0.6628	0.2456	0.2745	0.1432	0.1640	0.8360
rs34477782	0.4892	0.5108	0.2851	0.0722	0.7355	0.3202	0.3447	0.2459	0.7790	0.2210
rs4647655	0.3841	0.6159	0.3628	0.1607	0.9391	0.4676	0.4767	0.6808	0.6090	0.3910
rs3028822	0.4322	0.5678	0.3224	0.1007	0.8028	0.3772	0.4044	0.2109	0.2809	0.7191
rs2307783	0.4239	0.5761	0.3283	0.1074	0.8183	0.3890	0.4145	0.2431	0.7073	0.2927
rs10555216	0.3701	0.6299	0.3701	0.1607	0.9391	0.4676	0.4908	0.2957	0.5697	0.4303
rs10538061	0.6831	0.3169	0.1742	0.0210	0.5988	0.1650	0.1929	0.1105	0.8919	0.1081
rs10569275	0.5651	0.4349	0.2386	0.0474	0.6733	0.2574	0.2772	0.3181	0.1660	0.8340
rs35434967	0.5731	0.4269	0.2338	0.0454	0.6680	0.2515	0.2706	0.3324	0.8389	0.1611
rs3840222	0.6167	0.3833	0.2091	0.0343	0.6378	0.2161	0.2375	0.2577	0.8625	0.1375
rs3840794	0.4951	0.5049	0.2814	0.0696	0.7292	0.3143	0.3391	0.2372	0.7839	0.2161
rs11273905	0.3539	0.6461	0.3717	0.1334	0.8776	0.4303	0.4940	0.0040	0.5570	0.4430
rs36038238	0.4073	0.5927	0.3429	0.1321	0.8746	0.4283	0.4400	0.5955	0.6739	0.3261
rs34921138	0.6451	0.3549	0.1899	0.0315	0.6300	0.2063	0.2127	0.7252	0.8792	0.1208
rs146391383	0.7848	0.2152	0.1228	0.0081	0.5545	0.0982	0.1316	0.0260	0.9293	0.0707
rs3830479	0.6287	0.3713	0.2102	0.0252	0.6118	0.1827	0.2389	0.0030	0.1385	0.8615
rs10534050	0.3697	0.6303	0.3737	0.1733	0.9677	0.4833	0.4978	0.5116	0.5363	0.4637
rs5788637	0.4452	0.5548	0.3132	0.0891	0.7759	0.3556	0.3892	0.1198	0.7358	0.2642
rs147090496	0.3807	0.6193	0.3597	0.1416	0.8961	0.4420	0.4708	0.1939	0.3782	0.6218
rs16432	0.5346	0.4654	0.2592	0.0495	0.6787	0.2633	0.3064	0.0349	0.1886	0.8114
rs3835409	0.3830	0.6170	0.3527	0.1144	0.8344	0.4008	0.4576	0.0101	0.6464	0.3536
rs4147539	0.6016	0.3984	0.2145	0.0422	0.6593	0.2417	0.2445	0.8796	0.8576	0.1424
rs2307840	0.4422	0.5578	0.3203	0.1218	0.8512	0.4126	0.4009	0.5925	0.7230	0.2770
rs57406754	0.3707	0.6293	0.3736	0.1749	0.9714	0.4853	0.4977	0.5747	0.5373	0.4627
rs3216799	0.6703	0.3297	0.1742	0.0278	0.6192	0.1925	0.1929	0.9811	0.1081	0.8919
rs3044252	0.6392	0.3608	0.1922	0.0337	0.6363	0.2141	0.2156	0.9348	0.1228	0.8772
rs5896844	0.8992	0.1008	0.0538	0.0022	0.5258	0.0491	0.0554	0.5351	0.0285	0.9715
rs10580743	0.5672	0.4328	0.2347	0.0531	0.6878	0.2731	0.2719	0.9517	0.1621	0.8379
rs3842715	0.3798	0.6202	0.3699	0.1782	0.9788	0.4892	0.4905	0.9538	0.4293	0.5707
rs3034941	0.3841	0.6159	0.3628	0.1607	0.9391	0.4676	0.4767	0.6808	0.3910	0.6090
rs5788207	0.3991	0.6009	0.3748	0.2272	1.0923	0.5422	0.5002	0.0576	0.4872	0.5128
rs145119206	0.3923	0.6077	0.3539	0.1430	0.8993	0.4440	0.4599	0.4726	0.6424	0.3576
rs3047538	0.6862	0.3138	0.1717	0.0210	0.5988	0.1650	0.1899	0.1532	0.8939	0.1061
rs3033760	0.4451	0.5549	0.3155	0.1062	0.8157	0.3870	0.3929	0.7864	0.7318	0.2682

Note. MP: matching probability; PD: power of discrimination; PIC: polymorphic information content; PE: power of exclusion; TPI: typical paternity index; Ho: observed heterozygosity; He: expected heterozygosity; *P*: *P* values of HWE tests for 39 AIM-InDel loci; insertion: insertion allele frequencies; deletion: deletion allele frequencies.

## Data Availability

The data used to support the findings of this study are available from the corresponding author upon request.

## Supplementary Materials

Supplementary 1 Supplementary Table 1: the detail information of the reference populations in 1000 Genomes Project Phase 3.

Supplementary 2 Supplementary Table 2: the essential information and Rosenberg's *I*_n_ values of the 39 AIM-InDels.

Supplementary 3 Supplementary Table 3: the ancestry components of 27 populations calculated by STRUCTURE v2.3.4 based on raw genotype results of the same 38 InDels.
